# Suppression of peripheral NGF attenuates neuropathic pain induced by chronic constriction injury through the TAK1-MAPK/NF-κB signaling pathways

**DOI:** 10.1186/s12964-020-00556-3

**Published:** 2020-04-20

**Authors:** Wen-Ling Dai, Bing Yan, Yi-Ni Bao, Ji-Fa Fan, Ji-Hua Liu

**Affiliations:** 1 Jiangsu Key Laboratory of TCM Evaluation and Translational Research, School of Traditional Chinese Pharmacy, China Pharmaceutical University, Nanjing, 211198 Jiangsu China; 2 State Key Laboratory of Natural Medicines, China Pharmaceutical University, Nanjing, 210009 Jiangsu China

**Keywords:** *levo*-Corydalmine (*l*-CDL), Nerve growth factor (NGF), Neuropathic pain, Transforming growth factor-β-activated kinase 1 (TAK1), periphery

## Abstract

**Background:**

Anti-nerve growth factor (NGF) monoclonal antibodies (anti-NGF mAbs) have been reported to significantly attenuate pain, but the mechanism involved has not been fully elucidated, and the serious adverse events associated with mAbs seriously limit their clinical use. This study further investigated the mechanism by which peripheral NGF is involved in neuropathic pain and found safe, natural compounds that target NGF to attenuate neuropathic pain.

**Methods:**

Nociception was assessed by the Von Frey hair and Hargreaves’ methods. Western-blotting, qPCR and immunofluorescence were used to detect the cell signaling pathway. RAW264.7 macrophages and RSC96 Schwann cells were cultured for in vitro evaluation.

**Results:**

Intraplantar administration of anti-NGF mAbs suppressed the expression of phosphorylated transforming growth factor-β-activated kinase 1 (TAK1) in the dorsal root ganglion (DRG) and sciatic nerve. Intraplantar administration of a TAK1 inhibitor attenuated CCI-induced neuropathic pain and suppressed the expression of phosphorylated mitogen-activated protein kinases (MAPKs) in the DRG and sciatic nerve. Perisciatic nerve administration of *levo-*corydalmine (*l-*CDL) on the operated side obviously attenuated CCI-induced neuropathic pain and suppressed the expression of mNGF and proNGF. In addition, *l*-CDL-induced antinociception was reversed by intraplantar administration of NGF. Further results indicated that *l-*CDL-induced suppression of phosphorylated TAK1, MAPKs, and p65 and expression of the proinflammatory cytokines TNF-α and IL-1β in the DRG and sciatic nerve were all abolished by NGF. In addition, in vitro experiments indicated that *l-*CDL suppressed the secretion of NGF and proNGF in RAW264.7 macrophages and RSC96 Schwann cells, which was abolished by AP-1 and CREB agonists, respectively.

**Conclusions:**

This study showed NGF inhibition suppressed TAK1 in the periphery to attenuate CCI-induced neuropathic pain through inhibition of downstream MAPK and p65 signaling. The natural compound *l-*CDL inhibited NGF secretion by macrophages and Schwann cells and downstream TAK1-MAPK/NF-κB signaling in the periphery to attenuate CCI-induced neuropathic pain.

Video abstract

**Graphical abstract:**

Proposed mechanisms underlying the effect of *l*-CDL in periphery of CCI rats. In CCI rats, macropahages and Schwann cells could secret NGF to act on the receptors in the periphery to activate TAK1-MAPK/NF-κB axis and promote the release of proinflammatory cytokines, including TNF-α and IL-1β to promote neuropathic pain. *l*-CDL decreased the secretion of NGF through inhibiting AP-1 and CREB respectively in RAW264.7 and RSC96 Schwann cells to attenuate CCI-induced neuropathic pain by inhibiting the TAK1-p38 MAPK/NF-κB signaling pathway. 
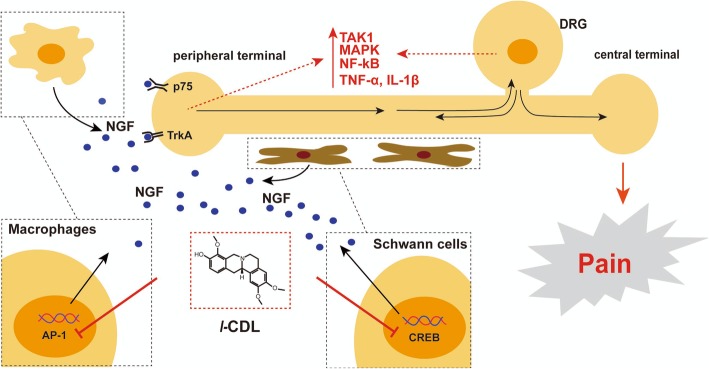

## Background

Approximately 6–8% of the population worldwide suffers from refractory neuropathic pain due to inadequate analgesia [1]. Opioids still dominate the clinical landscape despite their severe side effects. NGF and its interaction with its receptors have been well characterized as important mediators of pain initiation and maintenance in the periphery [2, 3]. Blocking NGF with mAbs or preventing NGF binding with its receptors effectively attenuates chronic pain, and anti-NGF mAbs such as tanezumab and fulranumab have been in clinical trials to attenuate chronic pain. However, in 2010, anti-NGF mAbs were placed on hold in all clinical trials by the FDA because of rapid joint destruction [4]. Although the FDA has since lifted this hold and a number of new trials are under way, the longterm efficacy and safety profile of anti-NGF antibodies have yet to be established. Elucidating the mechanism by which NGF is involved in neuropathic pain and finding safe drugs that target NGF to significantly attenuate chronic pain are promising.

After nerve injury, peripheral neuroglial Schwann cells and inflammatory cells such as macrophages are recruited to the lesion site and promote the secretion of NGF [5, 6]. The binding of NGF to its receptors activates several downstream signaling pathways, including MAPKs and nuclear factor-kappa B (NF-κB) signaling, to increase and sensitize various neurotransmitters, receptors and ion channels, including substance P, calcitonin gene-related peptide (CGRP), brain-derived neurotrophic factor (BDNF), bradykinin receptors, transient receptor potential vanilloid1 (TRPV1), and acid-sensing ion channels 2 and 3 (ASIC2 and 3), which contribute to neuropathic pain [2]. MAPKs, which include ERK1/2, JNK, p38, and NF-κB are crucial intracellular signaling pathways in chronic pain that mediate the transcription and synthesis of various inflammatory mediators and key transduction molecules including TNF-α and IL-1β, subsequently contributing to neuroinflammation and hypersensitivity [7]. TAK1, a member of the MAPKKK family, regulates various MAPK pathways. Inhibition of spinal TAK1 attenuates neuropathic pain [8], but whether peripheral TAK1 is involved and peripheral NGF-induced activation of MAPK was mediated by TAK1 have not yet been elucidated.

Our study hypothesized that NGF activates TAK1, thereby upregulating phosphorylated MAPKs to increase the release of proinflammatory cytokines and promote the development of CCI-induced neuropathic pain. In addition, finding safe and effective compounds to inhibit NGF and attenuate neuropathic pain would overcome the side effects of monoclonal antibody drugs. *l-*CDL is a trace alkaloid found in *Corydalis yanhusuo* W.T. Wang, which was found to have favorable antinociceptive effects in acute and chronic pain in our previous work, and the mechanism of *l-*CDL in the spinal cord has also been clarified [9–11]. However, the underlying mechanism of *l*-CDL in attenuating neuropathic pain in the periphery has still not been fully elucidated. Our results indicated that *l*-CDL decreased the expression of NGF mRNA and protein in the periphery to alleviate CCI-induced neuropathic pain, and we further evaluated the mechanism by which *l-*CDL inhibited NGF to regulate neuropathic pain suppression.

## Methods

### Ethics statement

All experimental protocols were approved by the Animal Experimentation Ethics Committee of China Pharmaceutical University and adhered to the guidelines of the International Association for the Study of Pain (IASP). All experiments were conducted in a blinded manner.

### Animals

Sprague-Dawley rats (180–220 g) were obtained from the Experimental Animal Center of Yangzhou University (Jiangsu, China). All animals were group-housed, six per cage, and acclimated to a temperature- and humidity-controlled environment with a 12:12 h light-dark cycle. Rodent chow and water were accessible ad libitum. Animals were randomly assigned to the following groups: 1) control; 2) CCI; 3) sham-operated; 4) CCI + Vehicle; 5) CCI + *l*-CDL (1.5, 3, and 6 mg/kg); or 6) CCI + NGF (0.1 μg/30 μL); 7) CCI + NGF (0.1 μg/30 μL) + *l*-CDL (6 mg/kg); 8) CCI + anti-NGF mAbs (6 μg/30 μL); or 9) CCI + TAK1 inhibitor (10 μg/30 μL). Six animals were assigned to each group for the behavior test, and four animals were assigned to each group for molecular testing.

### Chemicals and reagents

*l*-CDL (purity ≥99.0%, assessed by high performance liquid chromatography (HPLC)) was obtained from China Pharmaceutical University (Nanjing, China). Recombinant β-NGF and IL-1β were obtained from R&D Systems (Minneapolis, MN). Anti-NeuN antibody was purchased from Abcam (Cambridge, MA). Anti-NGF and anti-IL-1β antibodies were purchased from Santa Cruz Biotechnology (Santa Cruz, CA). Other primary antibodies and secondary antibodies for western blotting were obtained from Cell Signaling Technology (Beverly, MA). Secondary antibodies for immunofluorescence were obtained from Jackson ImmunoResearch Laboratories (Beverly, MA). Dulbecco’s modified Eagle’s medium (DMEM) was obtained from Biological Industries (Israel) and FBS was obtained from Gibco (Inc., PA). All other reagents were purchased from Sigma-Aldrich (St. Louis, MO). *l-*CDL was ground and dispersed in corn oil for animal experiment.

### Chronic constriction injury of the sciatic nerve

Chronic constriction injury of the sciatic nerve was conducted as described by Bennett and Xie [12]. All surgical instruments were disinfected in advance. The rats were anesthetized with pentobarbital (50 mg/kg, i.p.), and the left sciatic nerve was exposed. Four loose ligatures (4/0 catgut plain) were placed around the sciatic nerve at 1 mm intervals until a brief twitch in the hind paw was observed. For each ligature, we grasped the two ends close to the loop and tightened until the loop was just barely snug and the ligature did not slide along the nerve. We immediately stopped if a brief twitch was observed, to prevent arresting of epineural blood flow. Finally, the incision was sutured in layers. Penicillin was intramuscularly injected at a dose of 40,000 IU.

### Behavioral tests for pain states

The mechanical withdrawal threshold (MWT) and thermal withdrawal latency (TWL) were recorded for 2 days before surgery (day − 2, − 1). Behavioral tests were performed at 0, 0.5, 2, 4, 8, and 24 h after drug delivery on postoperative day 15. Each rat was tested three more times.

#### Mechanical allodynia

Von Frey hairs (Woodland Hills, Los Angeles) were used as previously described [10]. A series of Von Frey hairs with logarithmically incremental stiffness (1.4 ~ 15.0 g) were used to stimulate the hind paws of rats, and each hair was held for approximately 6 s. A positive response was defined as quick withdrawal or licking of the hind paw upon the stimulus. Whenever a positive response to a stimulus occurred, the next lower Von Frey hair was applied, and whenever a negative response occurred, the next higher hair was applied. Each rat was tested three more times, and the applied force (g) was recorded. Then the average of the threshold was determined to be the MWT.

#### Thermal hyperalgesia

Thermal hyperalgesia was determined by paw withdrawal latencies to radiant heat (model 37,370; Ugo Basile Biological Instruments). The radiant heat source was positioned at a fixed distance below the glass plate, and the left hind paw on the operated side was stimulated. The intensity of the light source was adjusted to produce withdrawal latencies of 10–13 s in control animals, which was 45 °C in our experiment, and the time until the rats licked and/or raised their foot was recorded. TWL was defined as the elapsed time (in seconds) between the delivery of the heat source and the withdrawal of the paw. A cutoff time of 25 s was set to prevent tissue damage.

### Cell culture

RSC96 and RAW264.7 cells were purchased from CHI Scientific, Inc. (Jiangyin, Jiangsu) and cultured in DMEM containing 10% v/v fetal bovine serum (FBS) at 37 °C in a humidified atmosphere containing 5% CO_2_. Both cells lines were plated in 6 well plates at 4 × 10^6^ cells per well and grown to approximately 80% confluence.

### Analysis of mRNA levels by quantitative real-time PCR (qPCR)

Total RNA was extracted using TRIzol reagent (Invitrogen Life Technologies, Carlsbad, CA, USA). RNA concentration was determined by a spectrophotometer at 260 nm and 280 nm. Equal amounts of RNA (1 μg) was reverse transcribed into cDNA, and the cDNAs were used as templates for PCR amplification. A QuantStudio 3 Real-Time PCR System and fast gene-expression method were used with the following cycling conditions: 95 °C for 5 min, followed by 45 cycles at 95 °C for 10 s, 57 °C for 20 s, and 72 °C for 20 s. Then, melt curve analysis was performed by raising the temperature from 61 °C to 95 °C at a rate of 0.15 °C/s. GAPDH was used as an internal control to normalize the variability in expression levels. The 2^−∆∆CT^ (cycle threshold) method was used to calculate the results, and mRNA expression levels are presented as fold-induction relative to Ctrl cells, which were set as 1. The specific primer sequences were as follows,

**Table:** 

NGF Forward 5′- CCAGTGAAATTAGGCTCCCTG − 3′ Reverse 5′- CCTTGGCAAAACCTTTATTGGG -3′ GAPDH Forward 5′- TGATGGGTGTGAACCACGAG − 3′ Reverse 5′- GCCCTTCCACAATGCCAAAG − 3′	

### Tissue collection and immunofluorescence

After 2 h of drug administration, the rats were anesthetized with pentobarbital (50 mg/kg, *i.p.*), and the ipsilateral L4-L6 DRGs and sciatic nerves were collected after the rats were perfused with PBS, followed by 4% paraformaldehyde. The tissues were postfixed with the same 4% paraformaldehyde and then transferred to 30% sucrose. Tissue sections were blocked with 10% normal donkey serum containing 0.3% Triton-X-100. After incubation, the tissue sections were observed under a laser-scanning microscope (Carl Zeiss LSM700, Germany). Eight images for each group were evaluated and photographed with the same exposure time to generate the raw data. Fluorescence intensities were analyzed using Image Pro Plus 6.0 (Media Cybernetics, Silver Spring, MD, USA), with *n* = 4 in each group.

### Western blotting

In brief, the rats were anesthetized with pentobarbital (50 mg/kg, i.p.) 2 h after drug administration, and the ipsilateral L4-L6 DRGs, and sciatic nerves were collected and extracted using RIPA buffer [10]. The proteins in the cell culture supernatants were extracted by methanol-chloroform precipitation [13]. The proteins were separated on sodium dodecyl sulfate-polyacrylamide gels, and transferred onto polyvinylidene difluoride membranes. After incubation with antibodies, immunoreactivity was detected using ECL reagents (PerkinElmer, Waltham, MA). The data were analyzed with the associated Quantity One-4.6.5 sofeware (Bio-Rad Laboratories), with *n* = 4 in each group.

### Statistical analysis

Sample size estimation was determined by G*Power 3.1 [14] and the powers (1-β err prob) were greater than 0.9 which was sufficient to detect differences between two different groups. All values are depicted as the mean ± SEM and the statistical analyses were performed using SPSS Rel 15 (SPSS Inc., Chicago, IL, USA). Data from western blotting, immunofluorescence and behavioral tests were statistically analyzed by one-way analysis of variance (ANOVA) and two-way ANOVA, followed by Bonferroni’s post-hoc tests with significance set at *P* < 0.05.

## Results

### Perisciatic nerve administration of *l-*CDL on the operated side attenuated CCI-induced neuropathic pain

**Fig. 1:**
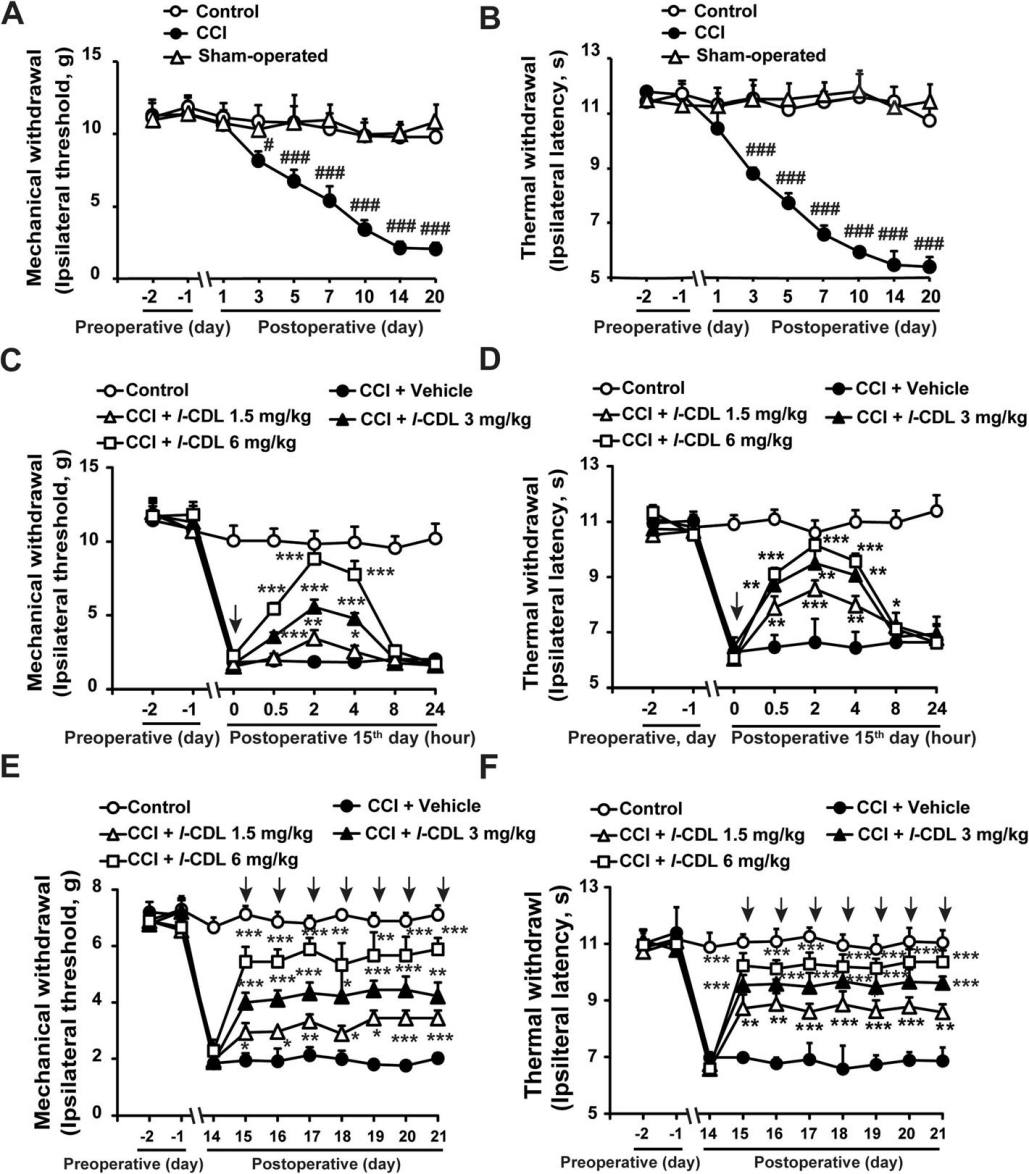
Perisciatic nerve administration of *l-*CDL on the operated side attenuated CCI-induced neuropathic pain. **a**, **b** Time course of the mechanical and thermal thresholds of SD rats at days 1, 3, 5, 7, 10, 14, and 20 after surgery compared with those of the control group. **c**, **d** Time course of the mechanical and thermal thresholds of SD rats after a single intramuscular injection of *l-*CDL (1.5, 3, and 6 mg/kg) around the muscles directly adjacent to the sciatic nerve on the operated side of CCI rats at day 15 after surgery (*n* = 6; *P < 0.05, **P < 0.01, and ***P < 0.001, compared with 0 h of each group; *#P* < 0.05, *##P* < 0.01, and *###P* < 0.001, compared with the control group). **e**, **f** Time course of the mechanical and thermal thresholds of SD rats after multiple injections of *l-*CDL (1.5, 3, and 6 mg/kg) around the muscles directly adjacent to the sciatic nerve on the operated side of CCI rats for 7 consecutive days. The mechanical and thermal thresholds were tested 2 h after *l-*CDL treatment each day (*n* = 6; *P < 0.05, **P < 0.01, and ***P < 0.001, compared with 14 d; #*P* < 0.05, and ##*P* < 0.01, compared with the control group)

The MWT and TWL of the injured paws of CCI rats were gradually reduced compared with those of the control group, and maintained a lower level on day 14 after surgery with no obvious difference between the control and Sham-operated groups (Fig. 1a, b). Perisciatic nerve administration of *l-*CDL (1.5, 3, and 6 mg/kg) ameliorated CCI-induced mechanical allodynia and thermal hyperalgesia in a dose- and time- dependent manner (Fig. 1c, d). The MWT and TWL of the saline control group, corn oil vehicle group and CCI group showed no obvious variation before or after administration. Moreover, consecutive injection of *l*-CDL (1.5, 3, and 6 mg/kg) for 7 days also showed definitive analgesia without tolerance or resistance (Fig. 1e, f).

### Perisciatic nerve administration of *l*-CDL suppressed the expression of NGF in the DRG and sciatic nerve of CCI rats

**Fig. 2:**
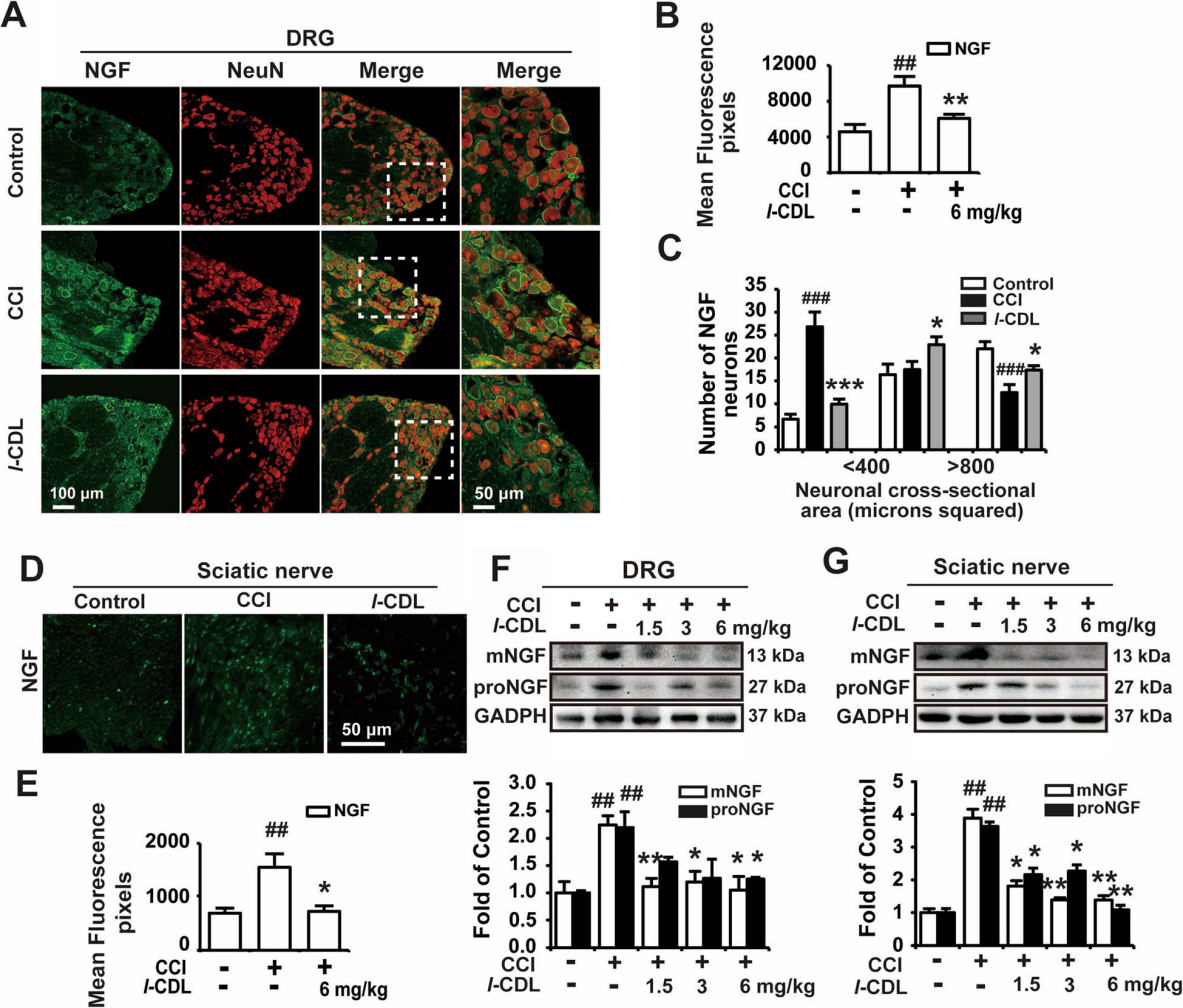
Perisciatic nerve administration of *l*-CDL suppressed the expression of NGF in the DRG and sciatic nerve of CCI rats. **a**, **b**, **d**, **e** Immunofluorescence results of intramuscular injection of *l-*CDL (6 mg/kg) around the sciatic nerve on the operated side of CCI rats showing the expression of NGF in the DRG and sciatic nerve. **c** The expression of NGF in different types of neurons after *l-*CDL (6 mg/kg) treatment in CCI rats. **f**, **g** Western blotting showing the expression of mNGF and proNGF in the DRG and sciatic nerve after *l-*CDL (1.5, 3, and 6 mg/kg) treatment of CCI rats. *n* = 4 per group; ##*P* < 0.01, and ###*P* < 0.001, compared with the control group; *P < 0.05, **P < 0.01, and ***P < 0.001, compared with the CCI group

NGF plays a vital role in neuropathic pain, and the high level of NGF in the periphery accelerates the development of peripheral sensitization and sympathetic budding [15]. The immunofluorescence results confirmed that NGF expression was increased in the DRG (Fig. 2a, b) and sciatic nerve (Fig. 2d, e) of CCI rats, and perisciatic nerve administration of *l-*CDL (6 mg/kg) decreased the upregulated expression of NGF in the DRG and sciatic nerve. DRG primary afferent neurons can be broadly classified as large (> 800), medium (400–800) and small (< 400) according to the cross sectional area of the neurons [16]. Aδ fibers have medium cell bodies and C fibers have small cell bodies that are nociceptors [17]. NGF was expressed mainly in the large and medium size neurons in normal rat DRGs. Our results indicated that in CCI rats, the expression of NGF in small neurons was notably increased. After *l*-CDL treatment, NGF was expressed mainly in the large and medium size neurons (Fig. 2a, c). Western blotting analysis also indicated that *l-*CDL (1.5, 3, and 6 mg/kg) inhibited the upregulated expression of mNGF and proNGF in the DRG (Fig. 2f) and sciatic nerve (Fig. 2g).

### Both *l-*CDL and anti-NGF mAbs inhibit the expression of p-TAK1 in the DRG and sciatic nerve, and *l-*CDL-induced antinociception and inhibition of p-TAK1 were reversed by intraplantar administration of NGF

**Fig. 3:**
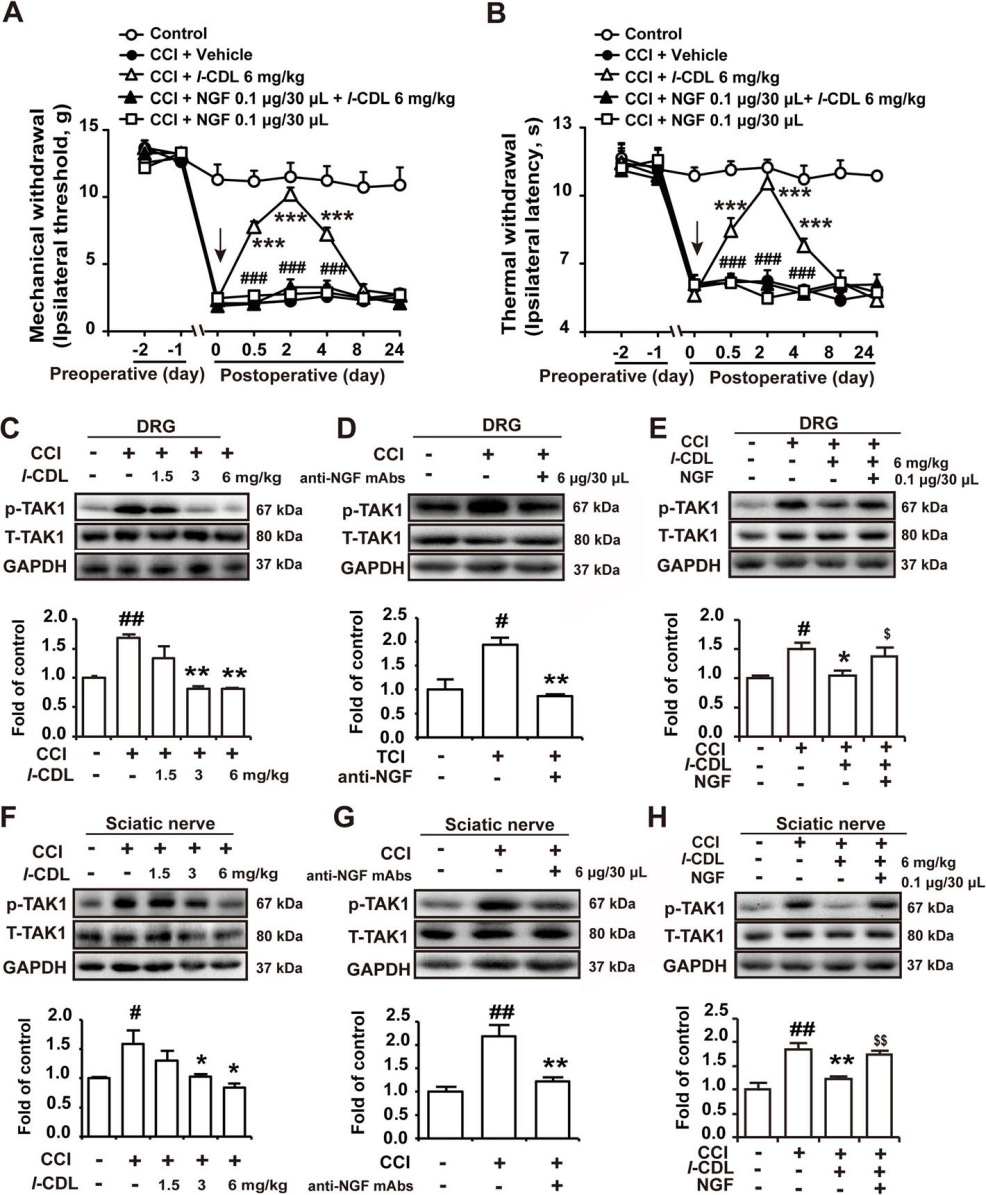
Both *l-*CDL and anti-NGF mAbs inhibited the expression of p-TAK1 in the DRG and sciatic nerve, and *l-*CDL induced antinociception and inhibition of p-TAK1 was reversed by intraplantar administration of NGF. **a**, **b** The mechanical and thermal thresholds of CCI rats after intraplantar administration of NGF (0.1 μg/30 μL) and intramuscular injection of *l-*CDL (6 mg/kg) around the sciatic nerve on the operated side of CCI rats (NGF was administered 15 min before *l-*CDL treatment). Vehicle: corn oil. *n* = 6 per group; ###*P* < 0.001, compared with *l-*CDL group; ***P < 0.001, compared with 0 h. The expression of p-TAK1 in DRG in CCI rats after intramuscular injection of *l-*CDL (6 mg/kg) around the sciatic nerve (**c**) and intraplantar administration of anti-NGF Abs (6 μg/30 μL) (**d**) or coadministration of *l-*CDL (6 mg/kg) with NGF (0.1 μg/30 μL) (**e**). The expression of p-TAK1 in the sciatic nerve in CCI rats after intramuscular injection of *l-*CDL (6 mg/kg) around the sciatic nerve (**f**) and intraplantar administration of anti-NGF Abs (6 μg/30 μL) (**g**) or coadministration of *l-*CDL (6 mg/kg) and NGF (0.1 μg/30 μL) (**h**). *n* = 4 per group; #*P* < 0.05, and ##*P* < 0.01, compared with the control group; *P < 0.05, and **P < 0.01, compared with the CCI group

Intraplantar administration of NGF (0.1 μg/30 μL) to the injured hind paw 15 min prior to *l*-CDL (6 mg/kg) injection completely abolished *l-*CDL-induced attenuation of mechanical (Fig. 3a) and thermal hyperalgesia (Fig. 3b), indicating that *l-*CDL induced antinociception was mediated by NGF. Intraplantar injection of NGF (0.1 μg/30 μL) alone in CCI rats had no significant difference compared with that of the CCI group. In addition, our results indicated that the expression of p-TAK1 was upregulated in both the DRG and sciatic nerve of CCI rats, and perisciatic nerve administration of *l-*CDL (1.5, 3, and 6 mg/kg) (Fig. 3c, f) and intraplantar administration of anti-mAbs (6 μg/30 μL) (Fig. 3d, g) also inhibited the upregulation of p-TAK1. And *l-*CDL-induced inhibition of p-TAK1 was also reversed by NGF (0.1 μg/30 μL) (Fig. 3e, h), suggesting that *l-*CDL induced inhibition of p-TAK1 was mediated by NGF.

### Intraplantar administration of the TAK1 inhibitor 5Z-7-oxozeaenol attenuated CCI-induced neuropathic pain and inhibited the upregulation of p-ERK, p-JNK, and p-p38 in the DRG and sciatic nerve

**Fig. 4:**
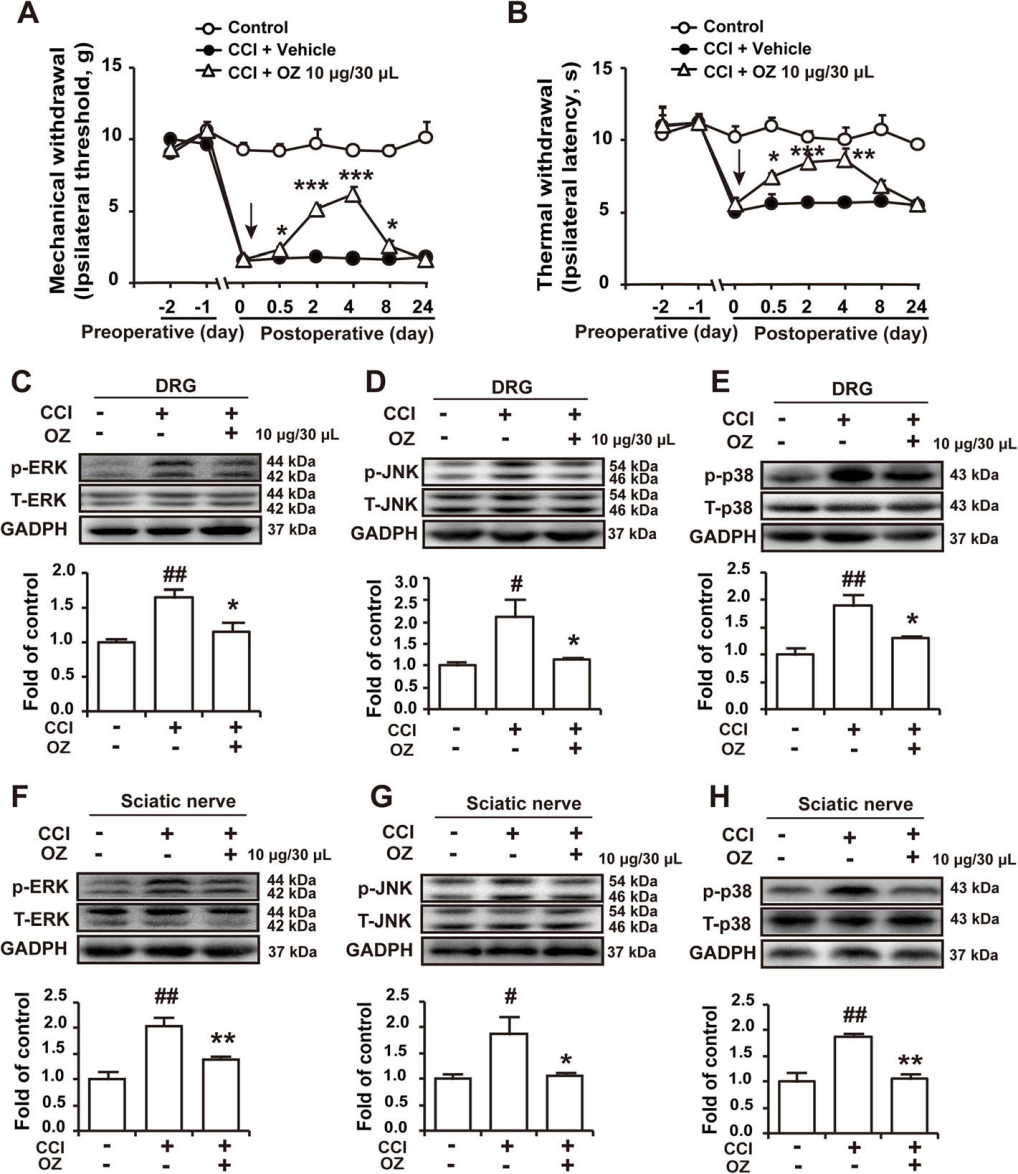
Intraplantar administration of the TAK1 inhibitor 5Z-7-oxozeaenol attenuated CCI-induced neuropathic pain and inhibited the upregulation of p-ERK, p-JNK, and p-p38 in the DRG and sciatic nerve. **a**, **b** The mechanical and thermal thresholds of CCI rats after intraplantar administration of OZ (10 μg/30 μL) on the operated side of CCI rats. Vehicle: 1% DMSO. n = 6 per group; #*P* < 0.05, ##*P* < 0.01, ###*P* < 0.001, compared with the *l-*CDL group; *P < 0.05, **P < 0.01, and ***P < 0.001, compared with 0 h. **c**-**e** The expression of p-ERK, p-JNK, and p-p38 in DRGs after intramuscular injection of the TAK1 inhibitor OZ (10 μg/30 μL). **f**-**g** The expression of p-ERK, p-JNK, and p-p38 in the sciatic nerve after intramuscular injection of the TAK1 inhibitor OZ (10 μg/30 μL). *n* = 4 per group; #*P* < 0.05, and ##*P* < 0.01, compared with the control group; *P < 0.05, and **P < 0.01, compared with the CCI group

Inhibition of spinal TAK1 has been reported to attenuate neuropathic pain, and we confirmed that intraplantar administration of the TAK1 inhibitor 5Z-7-oxozeaenol (OZ, 10 μg/30 μL) also attenuated CCI-induced neuropathic pain (Fig. 4a, b). In addition, intraplantar administration of the TAK1 inhibitor OZ inhibited the upregulation of p-ERK, p-JNK, and p-p38 in the DRG (Fig. 4c-e) and sciatic nerve (Fig. 4f-h) in CCI-induced neuropathic pain rats.

### *l-*CDL decreased the CCI-upregulated expression of p-ERK, p-JNK, p-p38, p-p65, TNF-α, and IL-1β in the DRG and sciatic nerve of CCI rats

**Fig. 5:**
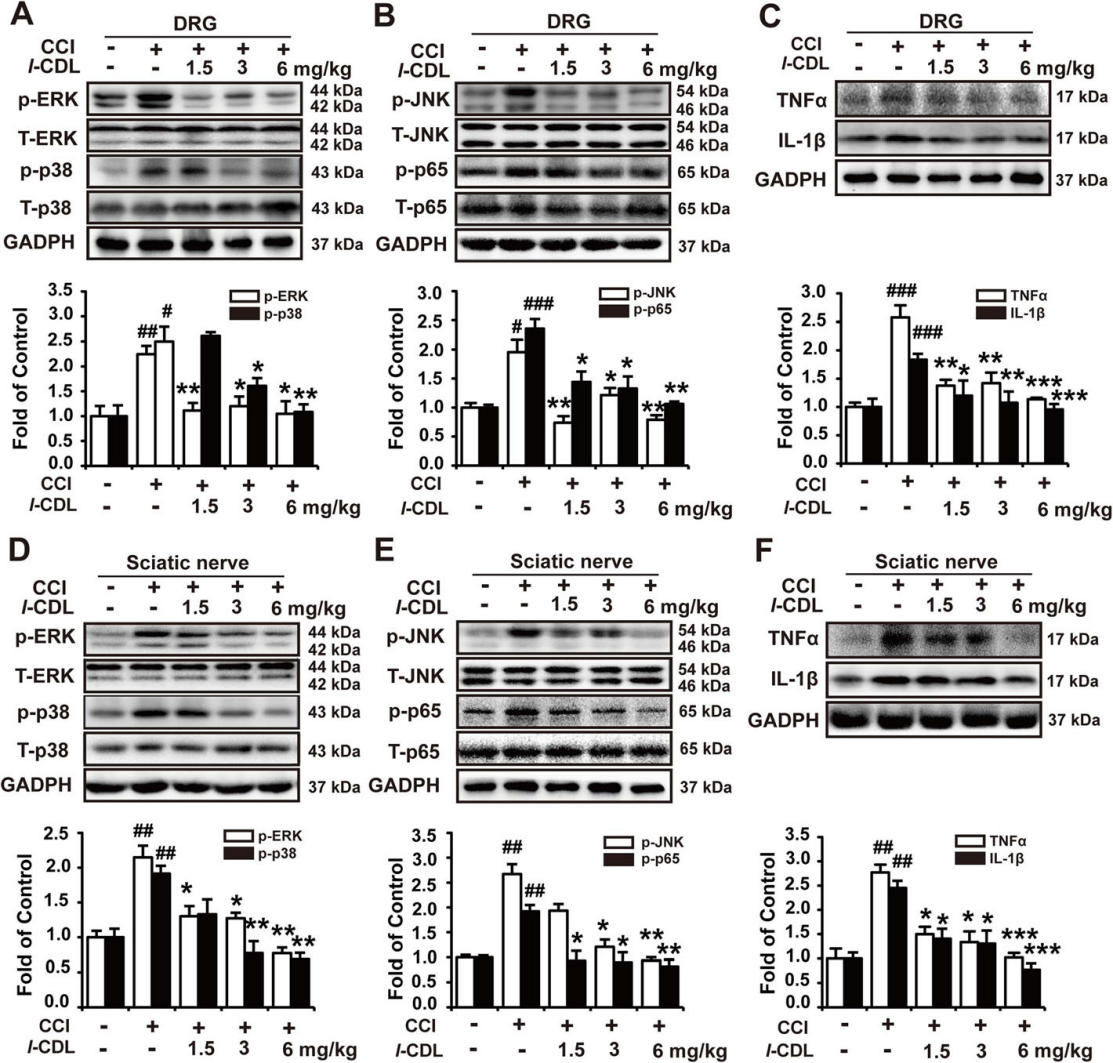
*l-*CDL decreased CCI-upregulated expression of p-ERK, p-JNK, p-p38, p-p65 and TNF-α, IL-1β in the DRG and sciatic nerve of CCI rats. The expression of p-ERK, p-p38 (**a**, **d**), p-JNK, p-p65 (**b**, **e**), TNF-α, and IL-1β (**c**, **f**) in the DRG and sciatic nerve in CCI rats after *l-*CDL (1.5, 3, 6 mg/kg) was intramuscularly injected around the sciatic nerve. n = 4 per group; #*P* < 0.05, ##*P* < 0.01, and ###*P* < 0.001, compared with the control group; *P < 0.05, **P < 0.01, and ***P < 0.001, compared with the CCI group

Sciatic nerve ligation evoked the inflammation in peripheral DRG and sciatic nerve in the ipsilateral of injured hind paw of CCI rats with high expression of inflammatory cytokines (such as TNFα, IL-1β) and activation of signaling molecules including JNK, ERK, p38, p65 contributing to peripheral hypersensitivity [18]. As depicted in Fig. 5, the expression of phosphorylated ERK, JNK, p38, and p65 and the inflammatory cytokines TNF-α and IL-1β were increased in the DRG (Fig. 5a-c) and sciatic nerve (Fig. 5d-f) of CCI rats. Perisciatic nerve administration of *l-*CDL (1.5, 3, and 6 mg/kg) on the operated side of CCI rats decreased the levels of TNF-α, and IL-1β and phosphorylated ERK, p38, JNK, and p65 both in the DRG (Fig. 5a-c) and sciatic nerve (Fig. 5d-f).

### Intraplantar administration of NGF reversed *l-*CDL-induced downregulation of p-JNK, p-ERK, p-p38, p-p65, TNF-α, and IL-1β in the DRG and sciatic nerve of CCI rats

**Fig. 6:**
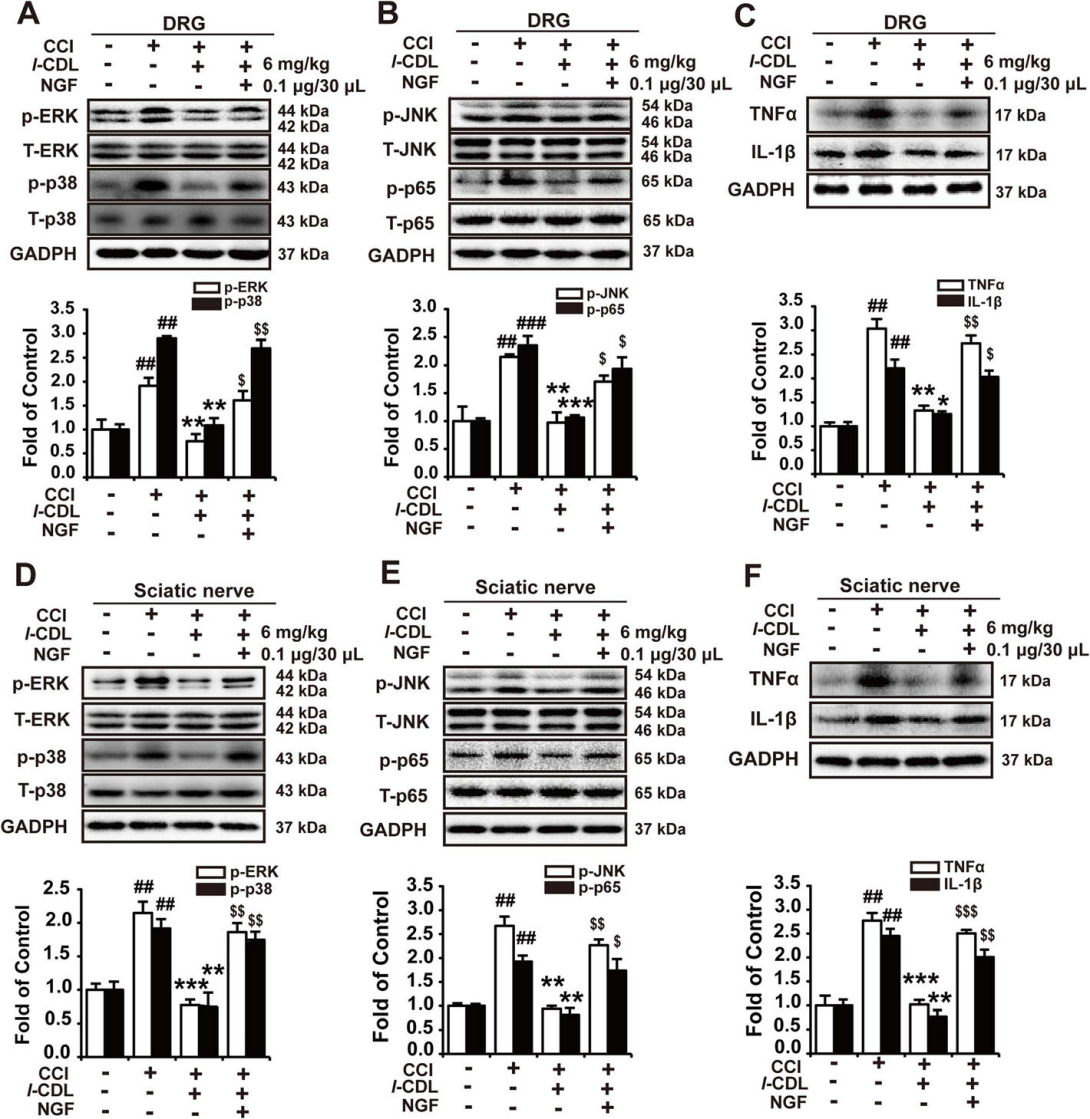
Intraplantar administration of NGF reversed *l-*CDL-induced downregulation of p-JNK, p-ERK, p-p38, p-p65, TNF-α, and IL-1β in the DRG and sciatic nerve of CCI rats. The expression of p-ERK, p-p38 (**a**, **d**), p-JNK, p-p65 (**b**, **e**), TNF-α, and IL-1β (**c**, **f**) in the DRG and sciatic nerve in CCI rats after coadministration of NGF (0.1 μg/30 μL) and *l-*CDL (6 mg/kg) (NGF was administered 15 min before *l*-CDL treatment). *n* = 4 per group; ##*P* < 0.01, and ###*P* < 0.001, compared with the control group; *P < 0.05, **P < 0.01, and ***P < 0.001, compared with the CCI group; $*P* < 0.05, $$*P* < 0.01, and $$$*P* < 0.001, compared with the *l-*CDL group

The upregulated expression of NGF could increase the expression of plenty of neurotransmitters and nociceptor-specific ion channels through activating ERK, p38, and JNK, NF-κB signaling [7, 15]. The results showed that *l*-CDL decreased the expression of p-JNK, p-ERK, p-p38, p-p65, TNF-α, and IL-1β in the DRG and sciatic nerve, and administration of NGF (0.1 μg/30 μL) completely abolished *l*-CDL (6 mg/kg)-induced inhibition of the expression of p-ERK, p-p38, p-JNK, p-p65, TNF-α, and IL-1β in the DRG (Fig. 6a-c) and sciatic nerve (Fig. 6d-f). These findings demonstrated the key role of p-ERK, p-p38, p-JNK, p-p65 signaling in the NGF regulated analgesia effect of *l*-CDL in the DRG and sciatic nerve.

### *l*-CDL inhibited AP-1 in macrophages and CREB in Schwann cells to decrease the transcription of NGF

**Fig. 7:**
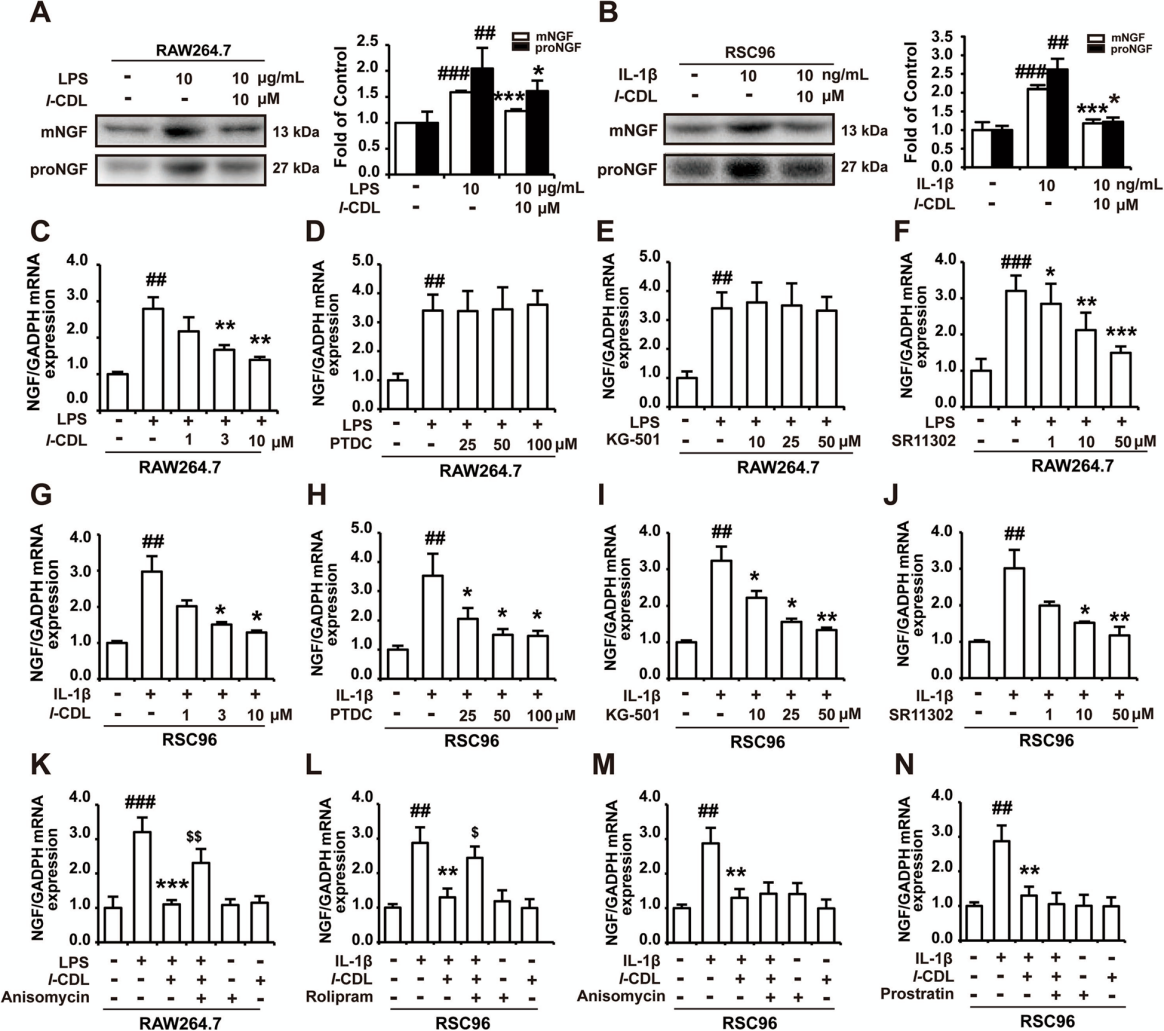
*l*-CDL inhibits AP-1 in macrophages and CREB in Schwann cells to decrease the transcription of NGF. **a** The expression of mNGF and proNGF after *l-*CDL (10 μM) treatment of RAW264.7 macrophages that were stimulated with LPS (10 μg/mL) for 12 h. **b** The expression of mNGF and proNGF after *l-*CDL (10 μM) treatment of RSC96 Schwann cells that were stimulated with IL-1β (10 ng/mL) for 12 h. **c** The mRNA level of NGF after *l-*CDL (1, 3, and 10 μM) treatment of RAW264.7 macrophages that were stimulated with LPS for 12 h. **g** The mRNA level of NGF after *l-*CDL (1, 3, and 10 μM) treatment of RSC96 Schwann cells that were stimulated with IL-1β for 2 h (*l-*CDL was administered 15 min before LPS and IL-1β treatment). **b-f** The mRNA level of NGF after treatment with the NF-κB inhibitor PDTC, CREB inhibitor KG-501, and AP-1 inhibitor SR11302 in RAW264.7 cells that were stimulated with LPS for 12 h. **h**-**j** The mRNA level of NGF after treatment with the NF-κB inhibitor PDTC, and CREB inhibitor KG-501, and AP-1 inhibitor SR11302 in RSC96 Schwann cells that were stimulated with IL-1β for 2 h. **k** The mRNA level of NGF after coadministration of the AP-1 activator anisomycin (30 μM) and *l-*CDL (10 μM) in RAW264.7 cells that were stimulated with LPS (10 μg/mL, 12 h). **l**-**n** The mRNA level of NGF after coadministration of the CREB agonist rolipram (20 μM), AP-1 activator anisomycin (30 μM), and NF-κB activator prostratin (5 μM) with *l-*CDL (10 μM) in RSC96 Schwann cells that were stimulated with IL-1β (10 ng/mL, 2 h) (all inhibitors or activators were administered 15 min resepectively before *l-*CDL treatment). *n* = 4 per group; #*P* < 0.05, ##*P* < 0.01, and ###*P* < 0.001, compared with the control group; *P < 0.05, **P < 0.01, and ***P < 0.001, compared with the CCI group; $*P* < 0.05, $$*P* < 0.01, and $$$*P* < 0.001, compared with the *l*-CDL group

Various reports indicate that NGF is prevailingly produced and secreted in non-neuronal cells, such as macrophages, schwann cells and fibroblast [6, 19–24]. The high level of IL-1β around the injury site strongly increases NGF in schwann cells and LPS was reported could promote NGF secretion in macrophage [25, 26]. Herein, LPS (10 μg/mL) and IL-1β (10 ng/mL) upregulated the protein expression of mNGF and proNGF in RAW264.7 macrophage and RSC96 Schwann cell supernatants. *l*-CDL (10 μM) significantly decreased the expression of mNGF and proNGF in both RAW264.7 macrophage (Fig. 7a) and RSC96 Schwann cell supernatants (Fig. 7b). LPS or IL-1β also upregulated the mRNA levels of NGF in RAW264.7 macrophages and RSC96 Schwann cells, and *l*-CDL (10 μM) significantly inhibited the upregulation of NGF mRNA in both RAW264.7 macrophages and RSC96 Schwann cells (Fig. 7c, g). The NF-κB inhibitor PDTC (25, 50, and 100 μM) and the CREB inhibitor KG-501 (10, 25, and 50 μM) did not inhibit LPS induced upregulation of NGF mRNA in RAW264.7 cells (Fig. 7d, e), while the AP-1 inhibitor SR11302 (1, 10, and 50 μM) obviously decreased the upregulation of NGF mRNA in RAW264.7 cells (Fig. 7f). The AP-1 activator anisomycin (30 μM) abolished *l-*CDL (10 μM)-induced inhibition of NGF mRNA expression in RAW264.7 cells (Fig. 7k). In RSC96 cells, the NF-κB inhibitor PDTC (25, 50, and 100 μM), CREB inhibitor KG-501 (10, 25, and 50 μM) and AP-1 inhibitor SR11302 (1, 10, and 50 μM) all inhibited IL-1β-induced upregulation of NGF mRNA (Fig. 7h-j), while only the CREB agonist rolipram (20 μM) but not the NF-κB agonist prostrain (5 μM) or AP-1 activator anisomycin (30 μM) abolished the *l*-CDL (10 μM)-induced decease in NGF mRNA (Fig. 7l-n).

## Discussion

In the present study, our results showed that inhibiting peripheral NGF suppressed TAK1, thereby inhibiting downstream ERK, p38, JNK, p65, TNF-α, and IL-1β to attenuate CCI-induced neuropathic pain. *l-*CDL also attenuated CCI-induced neuropathic pain by decreasing the expression of peripheral NGF and inhibiting the downstream pathway. Further study indicated that *l-*CDL decreased the transcription of NGF, thereby reducing the expression of NGF by inhibiting AP-1 and CREB in macrophages and Schwann cells, respectively, to reduce the synthesis and secretion of NGF.

Elevated NGF expression is observed in injured peripheral tissues, and inhibiting the expression of NGF reduces hyperalgesia in animal models of neuropathic pain and other pain states [2, 3]. Several approaches including blockade of NGF with mAbs, prevention of NGF binding to its receptors, and inhibition of TrkA function, have been used to attenuate chronic pain; among them, NGF mAbs have shown the greatest promise in clinical trials [2]. This might be because inhibitors blocking the binding of NGF to TrkA or inhibitors of TrkA both lack specificity to TrkA, as the TrkA family (TrkA, TrkB, and TrkC) has high homology with each other [27]. Several humanized anti-NGF mAbs have entered clinical trials, including tanezumab and fulranumab, but have been putted on hold by the FDA due to reports of destructive arthropathy [4]. Our research aimed to explore the mechanism of NGF inhibition induced antinociception and identify natural compounds that target NGF to attenuate neuropathic pain.

C*orydalis yanhusuo* W.T. Wang, a traditional Chinese medicine, has been widely used clinically as an analgesic for hundreds of years. Several natural products from *Corydalis yanhusuo* W.T. Wang have been demonstrated to have extensive pharmacological activities [28, 29]. In this report, our results indicated that perisciatic nerve administration of *l-*CDL (1.5, 3, and 6 mg/kg) on the operated side of CCI rats also attenuated CCI-induced neuropathic pain in a dose-dependent manner and without tolerance. Further results indicated that *l-*CDL decreased the upregulated expression of mNGF and proNGF in the DRG and sciatic nerve of CCI rats and that the antinociceptive effect of *l-*CDL was reversed by intraplantar administration of NGF to the injured hind paw. Interesting, in the DRG, *l-*CDL decreased NGF in small neurons and increased NGF in large and medium neurons. Small and medium cell bodies are nociceptors that transmit nociceptive stimuli, while medium and large cell bodies are nonnociceptive neurons [30]. NGF increases in small neurons, especially C fibers, with high TrkA intensity may promote neuropathic pain [31] by releasing SP and CGRP to promote central sensitization [32], indicating that *l-*CDL not only decreases the expression of NGF to exert antinociception but also maintains the physical function mediated by NGF in CCI rats. Our previous research also found that *l-*CDL shows affinity to both dopamine D1 receptors (D1DR) and dopamine D1 receptors (D2DR) with IC50 of 0.20 μM and 0.86 μM. And we found that *l-*CDL could antagonize spinal D1DR and D2DR to attenuate chronic pain (*the manuscript is being submitted*). In addition, *l-*CDL was also found in our previous study to alleviate chronic pain through inhibiting spinal N-Methyl-D-Aspartate (NMDA) and metabotropic glutamate1/5 (mGlu1/5) receptors [10]. But in the periphery, *l-*CDL was found showed great potential to suppress NGF, whether *l-*CDL induced inhibition of NGF was mediated by dopamine receptors or glutamate receptors will need further exploration. And our future research will investigate the relationships of dopamine receptors, glutamate receptors and NGF in the chronic pain. However, we also could not disavow the synergistic effects of *l-*CDL in multi-target approach, which might largely explain the strong observed effects in attenuating chronic pain.

NGF binding to its receptors was reported to activate MAPK family [33]. The MAPK family has three members, ERK, JNK, and p38, which promote the release of proinflammatory cytokines and are associated with chronic pain [34]. Activated MAPK contributes to NF-κB p65 activation, which promotes the production of proinflammatory cytokines such as TNF-α and IL-1β and leads to the development and maintenance of pain [35]. Our results found that *l*-CDL obviously inhibited the expression of p-ERK, p-p38, p-JNK, p-p65, TNF-α, and IL-1β in the DRG and sciatic nerve, which was reversed by NGF, indicating that *l-*CDL suppressed NGF to attenuate CCI-induced neuropathic pain though the inhibition of downstream MAPKs and the proinflammatory cytokines TNF-α and IL-1β. TAK1 is a member of MAPKKK family and is upstream of MAPK [36]. Inhibition of spinal TAK1 attenuates nerve injury-induced neuropathic pain [8]. While the expression of TAK1 in the periphery has not been clarified in neuropathic pain, we examined whether peripheral NGF-induced activation of MAPK was mediated by TAK1. Our results indicated that the expression of p-TAK1 was also upregulated in the DRG and sciatic nerve, and intraplantar administration of TAK1 inhibitor significantly attenuated CCI-induced neuropathic pain and inhibited the expression of MAPK in both the DRG and sciatic nerve. Both *l-*CDL and anti-NGF mAbs suppressed the expression of p-TAK1, and *l-*CDL-induced inhibition of p-TAK1 was reversed by NGF. In our research, *l-*CDL was found could attenuate CCI-induced neuropathic pain in the operated sides but not in controls. Our results indicated that *l-*CDL did not inhibit the expression of mNGF or proNGF in the DRG and sciatic nerve in control rats (data not shown) which might be different from the effect of NGF mAbs. And *l-*CDL induced inhibition of p-TAK1, p-MAPK, NF-kB and proinflammatory cytokines were all could be reversed by NGF, indicating that *l-*CDL inhibited the upregulated NGF in CCI rats and downstream TAK1-MAPK/NF-κB signaling to alleviate CCI-induced neuropathic pain.

Our results indicated that *l-*CDL inhibited the expression of both NGF and proNGF in the DRG and sciatic nerve. NGF is generated from proNGF, and tissue plasminogen activator is released by activated neurons to convert plasminogen into the active protease plasmin which ultimately cleaves proNGF to NGF [37]. NGF plays a vital role in neuropathic pain and is mainly synthesized and released by nonneuronal cells in the periphery compared with those of the spinal cord after damage to peripheral nerves.

Macrophages and Schwann cells are the main cells that synthesize proNGF in the periphery of rats. In this study, the results indicated that *l-*CDL decreased the mRNA levels of NGF, proNGF and NGF in RAW264.7 macrophages and RSC96 Schwann cells, indicating that *l*-CDL decreases NGF transcription, thereby reducing the expression of NGF to attenuate chronic pain. Previous reports indicated that NGF expression is mediated by heterodimeric AP-1 [38], NF-kB [39], and CREB [40]. In this study, *l-*CDL decreased the expression of NGF mRNA through AP-1 and CREB in RAW264.7 macrophages and RSC96 Schwann cells respectively. This was probably because of the different cell types. As mentioned above, in different cell types, the transcription factors that mediate NGF synthesis may be different.

## Conclusions

We provided the first experimental evidence that inhibiting peripheral NGF inhibits the TAK1-MAPK/NF-kB pathway to attenuate CCI-induced neuropathic pain. Perisciatic nerve administration of *l-*CDL on the operated side alleviated CCI-induced neuropathic pain by suppressing NGF secretion by macrophages and Schwann cells, thereby inhibiting TAK1 to inhibit downstream MAPK and NF-κB p65 signaling in the periphery. These results further explored the mechanism of NGF in neuropathic pain and confirmed that *l-*CDL is a potential therapeutic for pain management and offered pharmacological guidance for clinical application.

## Supplementary information

**Additional file 1.** Supplementary material

## Data Availability

The datasets used and/or analysed during the current study are available from the corresponding author on reasonable request.

## Abbreviations

NGF: Nerve growth factor
proNGF: precursor NGF
*l-*CDL: *levo-*Corydalmine
CCI: Chronic constriction injury; TAK1 transforming growth factor-β-activated kinase 1
Trk: Tropomyosin receptor kinase
DRG: Dorsal root ganglion
NF-κB p65: Nuclear factor light-chain-enhancer of activated B cells p65
CGRP: Calcitonin Gene-Related Peptide
BDNF: Brain-derived neurotrophic factor
TRPV1: Transient Receptor Potential Vanilloid1
ASIC2, 3: Acid-sensing ion channels 2 and 3
MWT: Mechanical withdrawal threshold
TWL: Thermal withdrawal latency
FBS: Fetal bovine serum
OZ: 5Z-7-oxozeaenol
GAPDH: Glyceraldehyde 3-phosphate dehydrogenase
LPS: Lipopolysaccharide
IL-1β: Interlukin-1 β
TNF-α: Tumor necrosis factor α
MAPK: Mitogen-activated protein kinase
ERK: Extracellular signal regulated kinase
JNK: c-Jun N-terminal kinase
CREB: cAMP response element binding protein
AP-1: Activating protein 1
FDA: US Food and Drug Administration
