# Supplementary material for: Suppression of peripheral NGF attenuates neuropathic pain induced by chronic constriction injury through the TAK1-MAPK/NF-κB signaling pathways
Source: Cell Commun Signal. 2020 Apr 20;18:66. doi: 10.1186/s12964-020-00556-3 (PMC7171864; doi:10.1186/s12964-020-00556-3)

Supplementary materials:

**Fig. 1** Western blotting showing the expression of mNGF and proNGF in the DRG and sciatic nerve after *l-*CDL (6 mg/kg) treatment of normal rats, n = 4 per group.


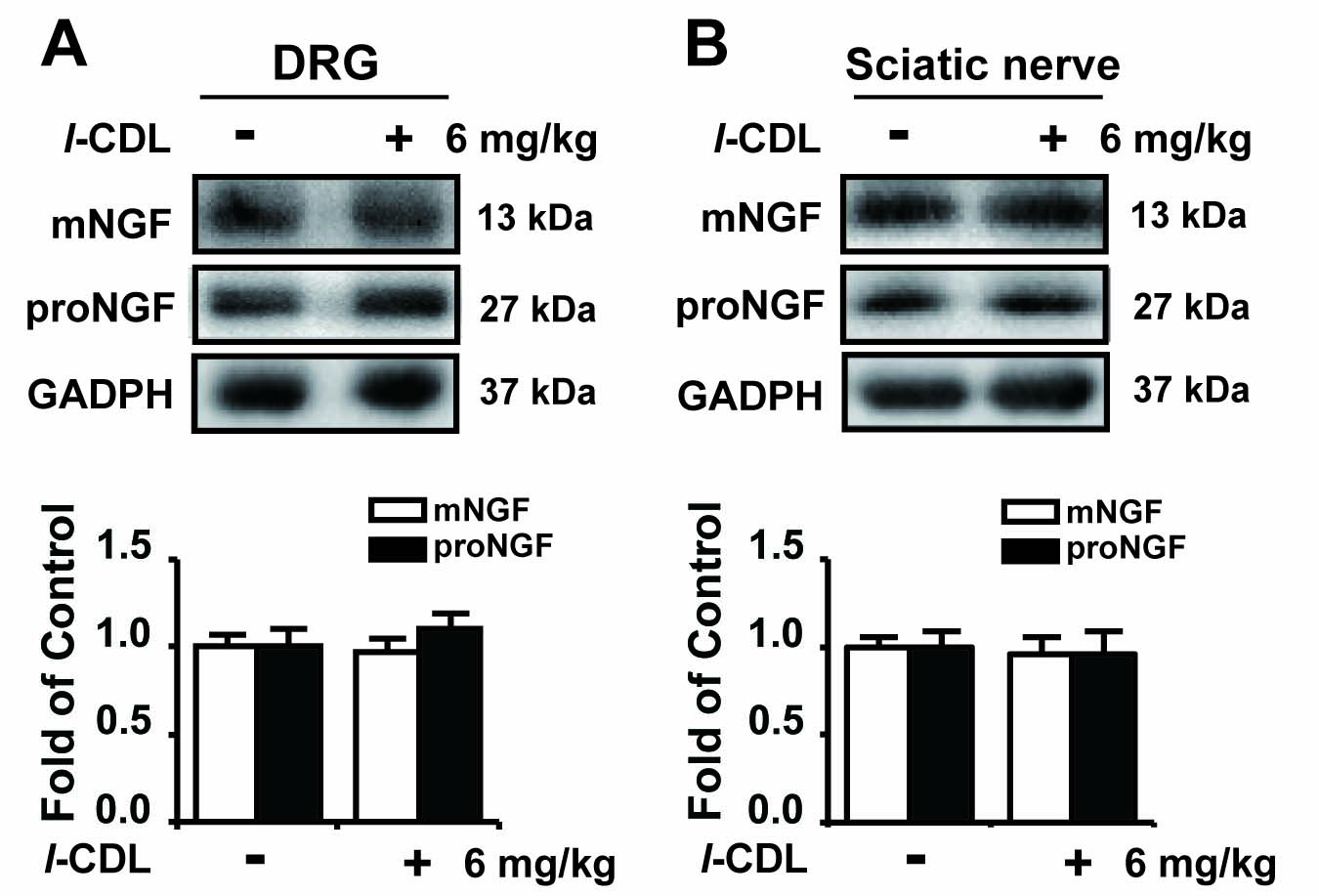

Supplement: Supplementary file 2 — Additional file 1. Supplementary material [file 12964_2020_556_MOESM2_ESM.docx]
